# An Overview of Adenoid Microbiome Using 16S rRNA Gene Sequencing-Based Metagenomic Analysis

**DOI:** 10.3390/medicina58070920

**Published:** 2022-07-11

**Authors:** Oļegs Sokolovs-Karijs, Monta Brīvība, Rihards Saksis, Gunta Sumeraga, Francesca Girotto, Renārs Erts, Jana Osīte, Angelika Krūmiņa

**Affiliations:** 1 Department of Otorhinolaryngology, Medical Faculty, Rīga Stradiņš University, 16 Dzirciema Str., LV-1007 Rīga, Latvia; gunta_sumeraga@inbox.lv; 2 AIWA Clinic, 241 Maskavas Str., LV-1019 Rīga, Latvia; 3 Latvian Biomedical Research and Study Centre, Rātsupītes Str. 1, LV-1067 Rīga, Latvia; monta@biomed.lu.lv (M.B.); rihards.saksis@biomed.lu.lv (R.S.); 4 Medical Faculty, Rīga Stradiņš University, Dzirciema Str., LV-1007 Rīga, Latvia; 042253@rsu.edu.lv; 5 Faculty of Medicine, Latvian University, Raina Blvd. 19, LV-1586 Riga, Latvia; renars.erts@gmail.com; 6 Centrālā Laboratorija, Šarlotes Str. 1b, LV-1011 Rīga, Latvia; jana.osite@laboratorija.lv; 7 Department of Infectology, Medical Faculty, Rīga Stradiņš University, 16 Dzirciema Str., LV-1007 Rīga, Latvia; krumina.angelika@inbox.lv

**Keywords:** adenoids, microbiome, bacteria, 16s rRNA gene sequencing

## Abstract

*Background and Objectives*: the upper respiratory tract harbors the highest bacterial density in the whole respiratory system. Adenoids, which are located in the *nasopharynx*, are a major site of bacterial colonies in the upper airways. Our goal was to use culture-independent molecular techniques to identify the breadth of bacterial diversity in the adenoid vegetations of children suffering from chronic rhinosinusitis and obstructive sleep apnea. *Materials and methods*: in total, 21 adenoid samples were investigated using amplification and sequencing of the V3-V4 hypervariable region of the bacterial 16S rRNA gene. *Results*: among the most common bacterial species found were *Veillonella atypica*, *Fusobactrium nucelatum*, *Shaalia odontolytica*, and *Moraxella catarrhalis*. *Veillonella atypica* and *Fusbacterium* *nucelatum* dominated the microbiome in all 21 samples, attributing to more than 60% of all detected genetic material. *Conclusions*: since both *Veillonella atypica* and *Fusobacterium nucleatum* are, predominantly, oral cavity and dental microorganisms, our findings may suggest oral microbiome migration deeper into the oropharynx and nasopharynx where these bacteria colonize adenoid vegetations.

## 1. Introduction

The upper respiratory tract of a human houses a community of commensal, symbiotic and pathogenic microorganisms. Furthermore, the upper respiratory tract harbors the highest bacterial density in the whole respiratory system [1]. These microorganisms play a critical role in the regulation of many homeostatic processes, including inflammation and immune response as well as defense against other, potentially more harmful pathogens [2]. An alteration in the composition of these microbial communities can lead to numerous pathological conditions, for example—infections [3]. 

Microbial communities congregate and attach themselves to biological and non-biological surfaces and produce their own matrix [4]. Most bacteria coexist in this state. A large major site of accumulation of these microorganisms is adenoid vegetations in the nasopharyngeal region of the upper airways [3]. Biofilms have been associated with the development and recurrence of different chronic upper-respiratory tract infections [5]. Moreover, bacteria in biofilm configuration result in reduced metabolic activity, adopting a dormant state that causes decreased susceptibility to antimicrobial agents, consequently contributing to the persistence of pathogens [6]. Many studies show that biofilm-associated resistance to antimicrobial agents is due to a significant delay in the penetration process into the biofilm extracellular matrix and to the decreased growth rate of the organisms [5]. 

The most common bacterial communities inhabiting the nose and throat of a healthy individual belong to the phyla *Firmicutes*, *Bacteroidetes*, *Actinobacteria*, and *Proteobacteria*, with a prevalence of genera *Bifidobacterium*, *Corynebacterium*, *Staphylococcus*, *Streptococcus*, *Haemophilus*, *Dolosigranulum* and *Moraxella*. The oropharynx, compared to the nasopharynx, presents with more diverse bacterial communities with a prevalence of *Streptococcal* species, *Neisseria* spp., *Rothia* spp., and anaerobes [7,8]. Detection of these microorganisms is essential for effective treatment of upper respiratory-tract infections. Different techniques, throughout the years, have been developed to isolate and research individual microorganisms on biological surfaces. Several infectious agents require being isolated in specialized liquid or solid media that allow the growth of specific species.

The most common agent used in solid artificial media is agar, which allows researchers to quantify and identify colonies [9]. For a long time, classic, agar-based bacterial isolation and growth techniques were the primary means of microbiological research.

An alternative approach is through genetic probes, which identify microorganisms by detecting unique DNA or RNA nucleotide sequences, focusing on specific target regions. 16S ribosomal RNA gene polymerase chain reaction is used to identify the composition of bacterial communities and compare their phylogeny and taxonomy in precise areas [10].

Studies investigating bacterial colonies of the adenoid surface have been conducted for multiple years, but it is only recently that the aforementioned modern 16s ribosomal RNA gene-sequencing technique started to get implemented. Studies using this method are few and show varied results, thus we believe we can contribute our own data and further explore the bacterial composition in the nasopharynx.

Our goal is to evaluate the bacterial microflora colonizing the adenoid surface using the aforementioned 16s ribosomal RNA gene-sequencing in contrast to the classic agar-based microbiological research and to compare our results to other similar studies (both 16s ribosomal RNA-based and gara-based studies).

## 2. Materials and Methods

### 2.1. Ethics Statement

The research is compliant with the Helsinki Declaration and is approved by Riga Stradins University ethics committee Nr 2-PĒK-4/264/2022. All participants sign an ethics committee approved informed consent.

### 2.2. Patient Selection, Inclusion and Exclusion Criteria

For our research, we used 21 samples of adenoid tissue collected during an adenectomy operation from children 3 to 6 years of age, and two negative control samples containing water. The setting of operation was the AIWA Clinic multifunctional, surgical outpatient clinic in Riga. The time of sample collection was from September to November 2021. All operations were completed under general anesthesia, and performed by certified surgeons, based on adenoid surgery indications.

#### 2.2.1. Patient Inclusion Criteria

- Adenoid tissue collected from patients 2.5 to 6 years old.
- Indications for adenoid surgery—obstructive sleep apnea symptoms, recurrent upper airway infection, recurrent middle ear infections.
- Operation under general anesthesia.
- No evidence of middle ear fluid—A-type tympanogram.
- Endoscopically confirmed adenoid tissue in the nasopharynx blocking the choanas and/or eustachian tube openings.

#### 2.2.2. Patient Exclusion Criteria

- Acute upper-airway infection during operation (including fever > 38.0 °C, purulent discharge from the nose, adenoid and tonsillar exudate, productive cough).
- Immunocompromised patients: HIV patients, diabetes mellitus patients, patients with tumors undergoing chemotherapy and/or radiotherapy.
- Patients with congenital cleft malformations.
- Patients receiving bacterial microflora-altering medications (for example -immune system stimulators).

### 2.3. Material Collection

Prior to the removal of adenoid tissue, the surgeon disinfects the skin around the mouth as well as the inner surface of teeth and buccal mucosa. During the process of adenoid surgery, the surgeon ensured there was no contact between the adenoid tissue surface and the mucosa of the mouth or the oropharynx, so that the microbiome of the adenoid surface remained uncontaminated by the bacteria of the adjacent structures. Samples needed for research were obtained using sterile swabs that were placed on the adenoid surface and multiple times rotated. Swab tips were then placed in ‘’COPAN-eNat’’ System for nucleic acid collection and preservation, and immediately transported to Latvian Biomedical Research and Study Centre for further processing and sequencing.

### 2.4. Sample Processing and Sequencing

Microbial DNA was extracted using FastDNA Spin Kit for Soil (MP Biomedicals, Santa Ana, CA, USA) according to the instructions of the manufacturer. FastPrep-24™ Instrument (MP Biomedicals, USA) was used for sample lysis.

The V3-V4 hypervariable region of the bacterial 16S rRNA gene was amplified using the 341F/805R set of primers. A unique set of oligonucleotides was added to each sample during the second amplification to ensure dual indexing (Table S1 in the attached materials). Two negative controls were introduced during the PCR amplification, evaluated by the agarose gel electrophoresis, and sequenced applying one negative control per each of two sample batches. Magnetic-bead-based purification (Macherey-Nagel, Düren, Germany) was performed for each sample after amplification. The quantity and quality of extracted DNA and amplicon libraries were determined by Qubit Fluorometer (Thermo Fisher Scientific, Waltham, MA, USA) and Agilent 2100 Bioanalyzer systems (Agilent, Santa Clara, CA, USA), respectively. Sequencing was undertaken on the MiSeq System (Illumina, San Diego, CA, USA) with MiSeq Reagent Kit v2 (500-cycles) (Illumina, USA) obtaining at least 100,000 paired-end sequencing reads per sample.

### 2.5. Data Analysis

We began by evaluating the sequencing quality of the data by using both FastQCand MultiQC software. No sequence or base-level quality trimming was performed at this point. Next, the data was imported into QIIME2, where we performed trimming of primer sequences to select reads matching the V3 and V4 hypervariable regions, as sequenced, which allowed us to remove any leftover adapter contamination as well. The amplicon sequencing data are available in the Europan Nucleotide Archive (accession number: PRJNA852532).

### 2.6. Sample Collection Limitations

Our sample size is limited to 21 subjects due to several limitations.

- Samples could only be collected from individuals (or their representatives) who agreed to paticipate in the study and signed a consent form. A significant amount of potential participants did not agree upon examining the consent form.
- To adequately perform a swab of the adenoid surface the adenoid tissue itself needs to be removed. Unlike swabs of other organic surfaces, which are easily accesible, a surgical operation is needed to collect adenoid surface swabs. This significantly limits our participant pool.
- Control groups consisting of “healthy’’ individuals, more precisely adenoid surface swabs of participants who have no previously mentioned symptoms is problematic. As stated, to acquire a uncontaminated adenoid surface swab the adenoid tissue needs to be removed, however, a healthy individual does not require an adenoid surgery. Another way to perform a swab of the adenoid surface is transnasaly, but this method has a high contamination risk. We should also keep in mind that the target population is children and any invasive procedures, such as swabs of areas of limited accessibility require complete anesthesia to prevent discomfort.

## 3. Results

In total, twenty-one samples were collected from the adenoid tissue of twenty-one patients. Out of twenty-one samples—11 were male patients’ samples and 10 were from female patients.

### 3.1. Alpha and Beta Diversity

**Figure 1:**
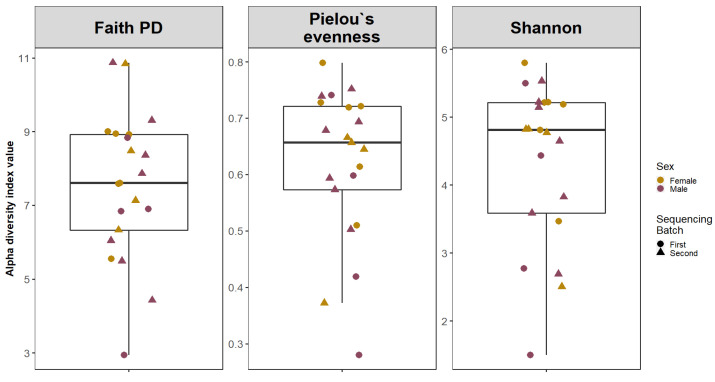
**Alpha Diversity of adenoid microbiota.** The boxplots are showing the median values and interquartile ranges of Faith’s phylogenetic index (Faith PD), Shannon diversity index and Pielou’s evenness index.

Alpha diversity analysis was conducted using Faith’s phylogenetic index (Faith PD), Shannon diversity index, Pielou’s evenness index (Figure 1). Median values of samples were calculated using the Wilcoxon rank sum test.

**Figure 2:**
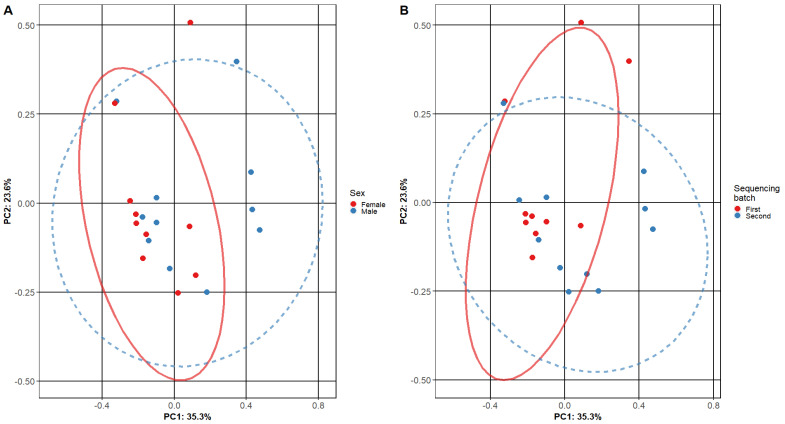
**Beta diversity of adenoid microbiome samples.** The plots show the composition of different adenoid microbiome sample subgroups: (**A**) comparing female and male subjects and (**B**) different sequencing batches. Each point represents the micro-biome signature of one sample, where different colors represent the various sample subgroups. The separation of samples is based on principal components, and the confidence level is set to 95% for ellipses. The overlap of ellipses in both figures indicates similar microbiome composition in the tested subgroups.

Beta diversity was calculated comparing female and male patient samples and two sequencing batch samples (Figure 2). A comparison of diversities of male and female patients shows a significant overlap between primary clusters (PC1 and PC2—male and female patient samples). Similar results were found when comparing our first and second sequencing batches.

### 3.2. Taxonomic Analysis

**Figure 3:**
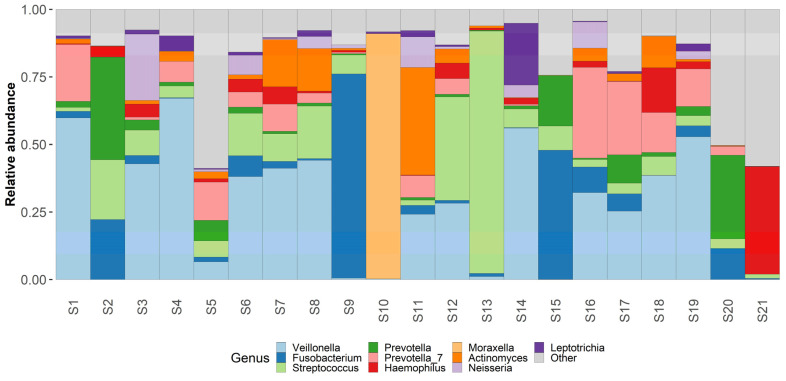
**Genus level taxonomy.** Taxaplot showing the relative abundances of most common genera within the samples.

**Figure 4:**
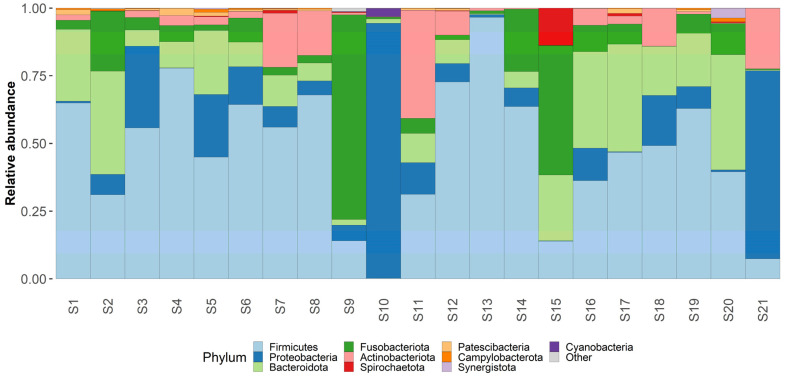
**Phylum level taxonomy.** Taxaplot showing the relative abundances of most common phyla within the samples.

Taxonomic analysis was conducted on both phylum and genus levels (Figure 3 and Figure 4) Phylum level analysis shows a significant relative abundance of firmicutes—42% (with an exception of two samples as seen in our taxonomic barplots). Firmicutes are followed by Proteobactria (18%), Bacteroidota (17%), Fusobacteriota (14%), and actinobacteriota (7%), other phyla occupy less than 1% each and can be found in the attached Table S1.

Genus level analysis shows an abundance of Veillonella (23%), Fusobacterium (11%), Streptococcus (11%), Prevotella (10%), Haemophilus (6%), and Moraxella (5%). Other non-table identified bacterial genus were Actinomyces, Leptotrichia, Neisseria, Klebsiella and Peptostreptococcus. Other identified bacterial genus can be found in the attached Table S1.

Regarding the most commonly identified bacterial species—*Fusobacterium nucleatum* had the highest relative abundance in our samples—18%. Other bacterial species with high abundance rates were *Veillonella atypica* (13%) and *Shaalia odontolytica* (7%), *Haemophylus influensae* (6%), *Prevotella shahii* (5%), and *Prevotella melaninogenica* (5%), *Streptococcus angiosus* (5%), *Prevotella intermedia* (4%), *Corynebacterium propinquum* (3%). Other identified bacterial species can be found in the attached Table S1.

## 4. Discussion

Even though a sample size of twenty-one samples may not be considered a large sample base, we must consider the fact that the adenoid surface is a location that is not easily reached. To obtain microbiological swabs without the risk of contamination the adenoids must be removed first, which significantly complicates sample collection and limits the amount of usable material.

As mentioned in our results, several samples had significantly different microbial communities, in comparison with other samples. As mentioned previously, the adenoid surface is hard to reach without removing the adenoids first and this anomaly in our results may be attributed to an error in sample collection. Even though the mouth cavity and the nasopharynx were not specifically disinfected prior to adenoid removal to minimize the risk of eradicating the inhibiting adenoid surface microflora, the skin around the mouth and the lips, as well as the inner surface of the teeth and buccal mucosa, were disinfected, according to surgical disinfection rules. Some amount of disinfectant may have come into contact with our adenoid samples before and during the removal process and tampered with the microflora of the surface.

Most studies mention *Haemophylus*, *Staphylococcus* and *Streptococcus* as the main microorganism colonizing the adenoid surface [11,12]. Our results show different microorganism domination—*Veillonela* and *Fusobacterium*. *Fusobacterium nucleatum* is an anaerobic oral commensal bacterium, mostly found in the oral mucosa. *Fusbactrium* primarily acts as a mutualist and is responsible for biofilm formation on the mucosa of the mouth, throat, and nasopharynx. *Fusobacterium* mostly contributes to gingival diseases and dental infections, while its role in extraoral infections remains unclear [13]. Some authors describe the identification of *Fusobactrium* in such pathological states as appendicitis, brain abscess, and osteomyelitis as co-infection, although *Fusobacterium* may not play a pivotal role in the pathogenesis [14]. The fact that *Fusobcaterium* was identified as the most common pathogen in our samples can indicate the extraoral migration of the microorganism towards the nasopharyx and the adenoid surface. Unfortunately, it may also indicate the flaws in our sample collection, specifically the contamination of the adenoid surface by buccal and dental microflora during adenoid removal through the mouth. More thorough disinfection of the oral cavity prior to tissue extraction by chlorohexidine or another antiseptic may reduce the risk of contamination, although excessive decontamination of the oral cavity may also disrupt the natural microbiome of the adenoids in the nasopharynx and lead to incorrect results.

Our second most commonly identified microorganism was *Veillonella atypica*—an oral cavity bacterium, commonly found in saliva, but is also found in the intestinal microbiome. *Veillonella atypica* abundance is linked to excessive caries development [15]. The fact that *Veillonella atypica* is also present in our samples of adenoid microflora can be described similarly to the abundance of *Fusobactrium nucleatum.* Either we observed the migration of *Veillonella atypica* from, predominantly, oral cavity and dental surfaces to the nasopharynx or from the flaws of our technique for removing adenoid tissue through the mouth, as discussed previously.

A study by Davcheva-Chakar et al. identified *Moraxella catarrhalis* as one of the most common bacteria found on the adenoid surface; the authors also report similar findings among other adenoid microbiome studies [16]. *Moraxella* genetic material was identified in our study, although not in a dominant position (5% of all genus-level microorganisms). This may contribute to other studies being conducted using samples from children with otitis media with effusion; our samples were from children with healthy middle ears. It is reported that *M. catarrhalis* co-infection with *Streptococcus pneumoniae* may promote *S. pneumoniae* to ascend the eustachian tube and cause the collection of fluid (either purulent or transudate) in the middle ear. Since our samples of the adenoid microbiome of children with healthy middle ears did not contain a dominant amount of either Moraxella or Streptococcus, this may indicate that these bacteria may play a role in the pathogenesis of otitis media.

*Moraxella catarrhalis* is also linked to adenoid and tonsillar hypertrophy. Studies investigating the abundance of this bacteria in adenotonsillar samples state that colonization by *Moraxella catarrhalis* may be associated with the development of more severe hypertrophy based on the assumption that this microorganism may cause severe inflammatory process in the tissue [17]. We did not include patients with enlarged palatine tonsils and, concurrently, our study included patients who had adenotomy operations without either tonsillotomy or tonsillectomy. This may indirectly indicate the role of *Moraxella catarrhalis* abundance in the process of palatine tonsil hypertrophy.

Lappan et al. reported a higher abundance of *Corynebacterium* and *Dolosigranulum* abundance in the adenoids of patients not suffering from otitis media with effusion [18]. We detected both *Dolosigranulum* genetic material and *Corynebacterium* on genus level although at a relatively low abundance. *Dolosigranulum* in our samples corresponds to studies that report colonization of the nasopharynx by *Corynebacterium* and *Dolosigranulum* species in children who received breastfeeding, as opposed to children who receive pre-made food supplements [19,20]. *Dolosigranulum* species have been linked in symbiosis with *Corynebactreum* species to nasal cavity microflora by Brueger et al. [21]. *Corynebactreum propinquum* was among the most commonly identified bacterial species, which in turn may indicate nasal microflora migration into the nasopharynx.

Biofilm production in the upper airways is linked with the development of chronic conditions, such as chronic rhinosinusitis, adenoiditis, and otitis media. *Staphylococcus aureus* was linked to biofilm formations [22]. Our results do not identify *Staphylococcus aureus*, or any other *Staphylococcus* species, as a significant component of bacterial microbiome of adenoids. Our findings may suggest a less significant role of *Staphylococcus* species in the formation of these conditions since our samples were collected from patients suffering from diseases such as chronic and intermittent rhinosinusitis, which are inherently biofilm-associated.

Two 16s RNA-based studies reported that *Firmicutes*, *Proteobacteria and Fusobacteria* were the most common microorganisms identified in the adenoid vegetation [23,24]. These results support our findings, because both *Veillonella*, *Streptococcus salivarius* and *Fusobactrium* combined attribute to more than 60% of microorganisms in our samples.

A study by de Morgado detected *Mycobactrium* leprae in their adenoid samples and reported it in their study. We did not identify this species of mycobacterium, although another species of Mycobacterium—*Mycobacterium Tuberculosis,* was identified. [25] The study itself was conducted in Brazil, which may explain this unexpected finding. Regarding the identified *M. tuberculosis*, Latvia is considered a region with, relatively, frequently diagnosed form of lung tuberculosis, which may explain traces of *M.tuberculosis* in our samples.

Other studies identify *Haemophilus influensae* as the most widespread taxa of bacteria found on the adenoid surface [26]. *H*. *Influensae* plays a significant role in the development of recurrent and chronic infection, mainly, in the pediatric population, rarely—in adults. *H. influensae* was also isolated in our samples, although to a lesser degree. This can be attributed to excessive governmental programs to promote *H. Influensae* vaccines in small children. Another reason why our data shows a much lesser *H. Influensae* concentration is that other studies used other methods of bacterial identification, such as conventional, agar-based methods.

To further elaborate on the role of adenoid microorganisms attributing to the development of middle-ear effusion, *Alloicoccus otitidis* is considered to be an organism found predominantly in the middle-ear fluid and in, relatively, low abundance on the adenoid surface [27,28]. This, partially, correlates with our results as no alloicocus genetic material was found whatsoever in our adenoid material from patients with no middle ear involvement. This may also indicate the role of not only *Moraxella* and *Streptococcus*, but also *Alloicoccus otitidis* in the pathogenesis of middle ear infections.

## 5. Conclusions

Bacterial colonies found on the adenoid surfaces of children suffering from recurrent upper-airway infections and obstructive sleep apnea mostly consist of oral and dental microflora (*Veillonella* species and *Fusobacterium* species) as well as a relative high abundance of both *Shaalia odontolytica and Haemophilus influensae*. These findings may suggest oral microflora migration into the nasopharynx. Practically no bacteria exclusive to the nasopharynx were identified in significant abundance. All identified microorganisms are also found in other anatomical locations in the upper airways.

We used twenty-one samples in our research. A sample pool of this size is comparable to other 16s rRNA microbiome studies; however, a larger sample size may, potentially, provide more a diverse overview of the microbiome of the adenoid surface.

Another field of further investigation is the microflora of the adenoid surface of children suffering from chronic middle-ear infections (not included in our study). A comparison between those two groups may provide new insight into the pathogenesis of chronic middle-ear infections.

## Data Availability

All data is available upon reasonable request from the corresponding author.

## Supplementary Materials

The following supporting information can be downloaded at: https://www.mdpi.com/article/10.3390/medicina58070920/s1, Table S1: List of oligonucleotides used for PCR amplification.

## References

[1] Man W.H., Peters W.A.A.D.S., Bogaert D. (2017). The microbiota of the respiratory tract: Gatekeeper to respiratory health. Nat. Rev. Genet..

[2] Buffie C.G., Pamer E.G. (2013). Microbiota-mediated colonization resistance against intestinal pathogens. Nat. Rev. Immunol..

[3] Marseglia G.L., Caimmi D., Pagella F., Matti E., Labó E., Licari A., Salpietro A., Pelizzo G., Castellazzi A.M. (2011). Adenoids during childhood: The facts. Int. J. Immunopathol. Pharmacol..

[4] Macassey E., Dawes P. (2008). Biofilms and their role in otorhinolaryngological disease. J. Laryngol. Otol..

[5] Donlan R.M. (2002). Biofilms: Microbial life on surfaces. Emerg. Infect. Dis..

[6] Hoa M., Syamal M., Schaeffer M.A., Sachdeva L., Berk R., Coticchia J. (2010). Biofilms, and chronic otitis media: An initial exploration into the role of biofilms in the pathogenesis of chronic otitis media. Am. J. Otolaryngol..

[7] Proctor D.M., Relman D.A. (2017). The Landscape Ecology and Microbiota of the Human Nose, Mouth, and Throat. Cell Host Microbe.

[8] Kumpitsch C., Koskinen K., Schöpf V., Moissl-Eichinger C. (2019). The microbiome of the upper respiratory tract in health and disease. BMC Biol..

[9] Washington J.A., Baron S. (1996). Principles of Diagnosis. Medical Microbiology.

[10] Sontakke S., Cadenas M.B., Maggi R.G., Diniz P.P.V., Breitschwerdt E.B. (2008). Use of broad range16s rdna PCR in clinical microbiology. J. Microbiol. Methods.

[11] Kim S.K., Hong S.J., Pak K.H., Hong S.M. (2019). Analysis of the Microbiome in the Adenoids of Korean Children with Otitis Media with Effusion. J. Int. Adv. Otol..

[12] Subtil J., Rodrigues J.C., Reis L., Freitas L., Filipe J., Santos A., Macor C., Duarte A., Jordao L. (2017). Adenoid bacterial colonization in a paediatric population. Eur. Arch. Oto-Rhino-Laryngol..

[13] Brennan C.A., Garrett W.S. (2019). Fusobacterium nucleatum—Symbiont, opportunist, and oncobacterium. Nat. Rev. Microbiol..

[14] Heckmann J.G., Lang C.J., Hartl H., Tomandl B. (2003). Multiple brain abscesses caused by Fusobacterium nucleatum treated conservatively. Can. J. Neurol. Sci..

[15] Brook I. (1996). Veillonella infections in children. J. Clin. Microbiol..

[16] Davcheva-Chakar M., Kaftandzhieva A., Zafirovska B. (2015). Adenoid Vegetations—Reservoir of Bacteria for Chronic Otitis Media with Effusion and Chronic Rhinosinusitis. PRILOZI.

[17] Prates M., Tamashiro E., Proenca-Modena J.L., Criado M.F., Saturno T.H., Oliveira A.S., Buzatto G.P., Jesus B., Jacob M.G., Carenzi L.R. (2018). The Relationship between Colonization by Moraxella catarrhalis and Tonsillar Hypertrophy. Can. J. Infect. Dis. Med. Microbiol..

[18] Lappan R., Imbrogno K., Sikazwe C. (2018). A microbiome case-control study of recurrent acute otitis media identified potentially protective bacterial genera. BMC Microbiol..

[19] Charbonneau M.R., Blanton L.V., DiGiulio D.B., Relman D.A., Lebrilla C.B., Mills D.A., Gordon J.I. (2016). A microbial perspective of human developmental biology. Nature.

[20] Bosch A.A., Levin E., Van Houten M.A., Hasrat R., Kalkman G., Biesbroek G., Piters W.A.A.D.S., De Groot P.-K.C., Pernet P., Keijser B.J. (2016). Development of Upper Respiratory Tract Microbiota in Infancy is Affected by Mode of Delivery. EBioMedicine.

[21] Brugger S.D., Eslami S.M., Pettigrew M.M., Escapa I.F., Henke M.T., Kong Y., Lemon K.P. (2020). Dolosigranulum pigrum Cooperation and Competition in Human Nasal Microbiota. mSphere.

[22] Stoodley P., Nistico L., Johnson S., Lasko L.A., Baratz M., Gahlot V., Ehrlich G.D., Kathju S. (2008). Direct demonstration of viable Staphylococcus aureus biofilms in an infected total joint arthroplasty. A case report. J. Bone Jt. Surg. Am. Vol..

[23] Liu C.M., Cosetti M.K., Aziz M., Buchhagen J.L., Contente-Cuomo T.L., Price L.B., Keim P., Lalwani A.K. (2011). The Otologic MicrobiomeA Study of the Bacterial Microbiota in a Pediatric Patient With Chronic Serous Otitis Media Using 16SrRNA Gene-Based Pyrosequencing. Arch. Otolaryngol. Head Neck Surg..

[24] Ren T., Glatt D.U., Nguyen T.N., Allen E.K., Early S.V., Sale M., Winther B., Wu M. (2013). 16S rRNA survey revealed complex bacterial communities and evidence of bacterial interference on human adenoids. Environ. Microbiol..

[25] Morgado de Abreu M.A.M., Nai G.A., Molina J.D., Gomes R.T., Paula N., Roselino A.M. (2020). Mycobacterium leprae on Palatine Tonsils and Adenoids of Asymptomatic Patients, Brazil. Emerg. Infect. Dis..

[26] Fekete-Szabo G., Berenyi I., Gabriella K., Urban E., Nagy E. (2010). Aerobic and anaerobic bacteriology of chronic adenoid disease in children. Int. J. Pediatric Otorhinolaryngol..

[27] Frank D.N., Spiegelman G.B., Davis W., Wagner E., Lyons E., Pace N.R. (2003). Culture-independent molecular analysis of microbial constituents of the healthy human outer ear. J. Clin. Microbiol..

[28] Jervis-Bardy J., Rogers G.B., Morris P.S., Smith-Vaughan H.C., Nosworthy E., Leong L.E.X., Smith R.J., Weyrich L.S., De Haan J., Carney A.S. (2015). The microbiome of otitis media with effusion in Indigenous Australian children. Int. J. Pediatr. Otorhinolaryngol..

