# Supplementary material for: An Overview of Adenoid Microbiome Using 16S rRNA Gene Sequencing-Based Metagenomic Analysis
Source: Medicina (Kaunas). 2022 Jul 11;58(7):920. doi: 10.3390/medicina58070920 (PMC9318310; doi:10.3390/medicina58070920)
Supplement: Supplementary file 1 [file medicina-58-00920-s001.zip › medicina-1758294-supplementary.pdf]

medicina

1. Supplementary material

Table S1. List of oligonucleotides used for PCR amplification.

Oligonucleotides used for the amplification of the V3-V4 hypervariable region of the bacterial 16S rRNA gene (1st PCR amplification)	
ID	Sequence
ci5_16S_V3_Fw (341F)	5’-TCGTCGGCAGCGTCAGATGTGTATAAGAGACAGNNNNNNCCTACGGGNGGCWGCAG-3’
ci7_16S_V4_Rs(805R)	5’-GTCTCGTGGGCTCGGAGATGTGTATAAGAGACAGNNNNNNGACTACHVGGGTATCTAATCC-3’
**Oligonucleotides used for indexing (2nd PCR amplification)**	
Name	Sequence
i7_Adapter_N701	5’-CAAGCAGAAGACGGCATACGAGATTAAGGCGAGTCTCGTGGGCTCGGAGATGTGTATAAGAGACAG-3’
i7_Adapter_N702	5’-CAAGCAGAAGACGGCATACGAGATCGTACTAGGTCTCGTGGGCTCGGAGATGTGTATAAGAGACAG-3’
i7_Adapter_N703	5’-CAAGCAGAAGACGGCATACGAGATAGGCAGAAGTCTCGTGGGCTCGGAGATGTGTATAAGAGACAG-3’
i5_Adapter_E501	5’-AATGATACGGCGACCACCGAGATCTACACTAGATCGCTCGTCGGCAGCGTCAGATGTGTATAAGAGACAG-3’
i5_Adapter_E502	5’-AATGATACGGCGACCACCGAGATCTACACCTCTCTATTCGTCGGCAGCGTCAGATGTGTATAAGAGACAG-3’
i5_Adapter_E503	5’-AATGATACGGCGACCACCGAGATCTACACTATCCTCTTCGTCGGCAGCGTCAGATGTGTATAAGAGACAG-3’
i5_Adapter_E504	5’-AATGATACGGCGACCACCGAGATCTACACAGAGTAGATCGTCGGCAGCGTCAGATGTGTATAAGAGACAG-3’
i5_Adapter_E505	5’-AATGATACGGCGACCACCGAGATCTACACGTAAGGAGTCGTCGGCAGCGTCAGATGTGTATAAGAGACAG-3’
i5_Adapter_E506	5’-AATGATACGGCGACCACCGAGATCTACACACTGCATATCGTCGGCAGCGTCAGATGTGTATAAGAGACAG-3’
i5_Adapter_E507	5’-AATGATACGGCGACCACCGAGATCTACACAAGGAGTATCGTCGGCAGCGTCAGATGTGTATAAGAGACAG-3’

i5_Adapter_E508	5'-AATGATACGGCGACCACCGAGATCTACACCTAAGCCTTCGTCGGCAGCGTCAGATGTGTATAAGAGACAG-3'
